# Sequence-dependent dynamics of synthetic and endogenous RSSs in V(D)J recombination

**DOI:** 10.1093/nar/gkaa418

**Published:** 2020-05-25

**Authors:** Soichi Hirokawa, Griffin Chure, Nathan M Belliveau, Geoffrey A Lovely, Michael Anaya, David G Schatz, David Baltimore, Rob Phillips

**Affiliations:** Department of Applied Physics, California Institute of Technology, Pasadena, CA 91125, USA; Division of Biology and Biological Engineering, California Institute of Technology, Pasadena, CA 91125, USA; Division of Biology and Biological Engineering, California Institute of Technology, Pasadena, CA 91125, USA; National Institute on Aging, National Institutes of Health, Baltimore, MD 21224, USA; Division of Biology and Biological Engineering, California Institute of Technology, Pasadena, CA 91125, USA; Department of Immunobiology, Yale University School of Medicine, New Haven, CT 06520, USA; Division of Biology and Biological Engineering, California Institute of Technology, Pasadena, CA 91125, USA; Division of Biology and Biological Engineering, California Institute of Technology, Pasadena, CA 91125, USA; Department of Physics, California Institute of Technology, Pasadena, CA 91125, USA

## Abstract

Developing lymphocytes of jawed vertebrates cleave and combine distinct gene segments to assemble antigen–receptor genes. This process called V(D)J recombination that involves the RAG recombinase binding and cutting recombination signal sequences (RSSs) composed of conserved heptamer and nonamer sequences flanking less well-conserved 12- or 23-bp spacers. Little quantitative information is known about the contributions of individual RSS positions over the course of the RAG–RSS interaction. We employ a single-molecule method known as tethered particle motion to track the formation, lifetime and cleavage of individual RAG–12RSS–23RSS paired complexes (PCs) for numerous synthetic and endogenous 12RSSs. We reveal that single-bp changes, including in the 12RSS spacer, can significantly and selectively alter PC formation or the probability of RAG-mediated cleavage in the PC. We find that some rarely used endogenous gene segments can be mapped directly to poor RAG binding on their adjacent 12RSSs. Finally, we find that while abrogating RSS nicking with Ca^2+^ leads to substantially shorter PC lifetimes, analysis of the complete lifetime distributions of any 12RSS even on this reduced system reveals that the process of exiting the PC involves unidentified molecular details whose involvement in RAG–RSS dynamics are crucial to quantitatively capture kinetics in V(D)J recombination.

## INTRODUCTION

Jawed vertebrates call upon developing lymphocytes to undergo a genomic cut-and-paste process known as V(D)J recombination, where disparate gene segments that do not individually code for an antigen–receptor protein are systematically combined to assemble a complete, antigen receptor-encoding gene (1). V(D)J recombination supports the production of a vast repertoire of antibodies and T-cell receptors that protect the host organism from a broad array of pathogens. However, gene segment combinations are not made in equal proportions; some gene segment combinations are produced more frequently than others (2–5). Although V(D)J recombination requires careful orchestration of many enzymatic and regulatory processes to ensure functional antigen–receptor genes whose products do not harm the host, we strip away these factors and focus on the initial stages of V(D)J recombination. Specifically, we investigate how the dynamics between the enzyme that carries out the cutting process and its corresponding DNA-binding sites adjacent to the gene segments influence the initial stages of recombination for an array of synthetic and endogenous binding site sequences.

**Figure 1.**
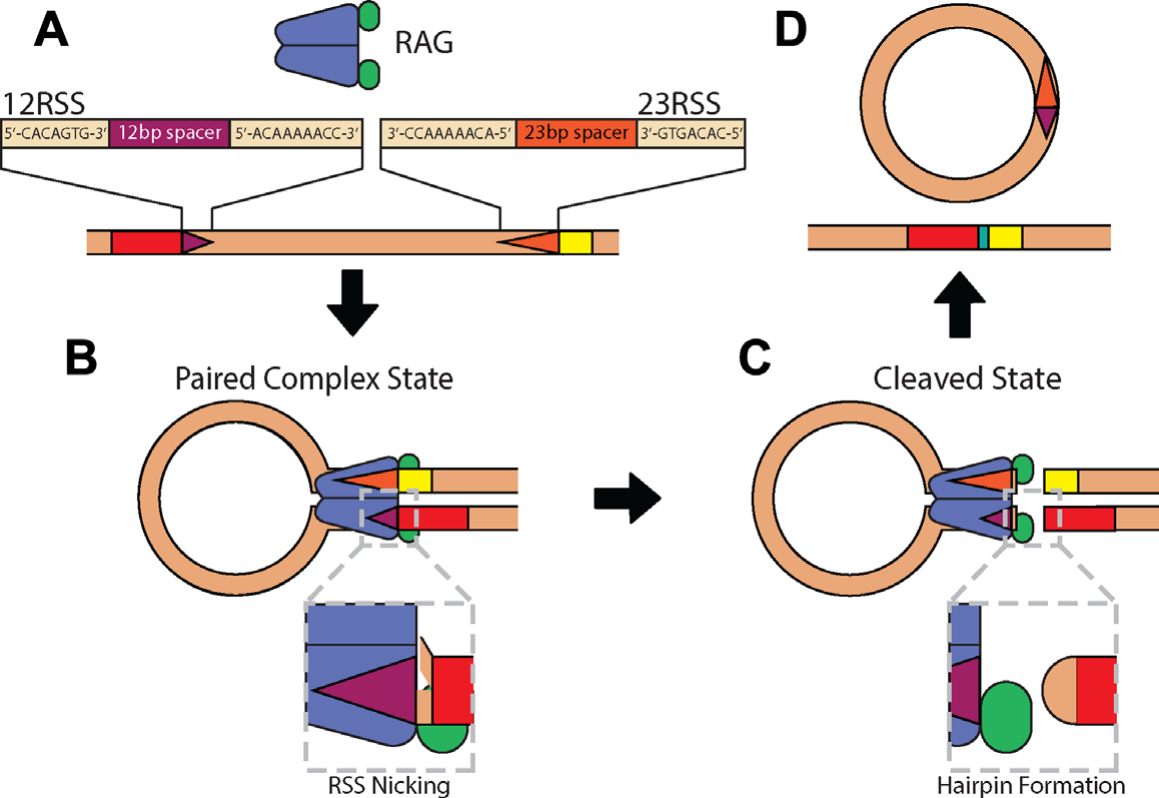
Schematic focusing on the initial steps of V(D)J recombination. (**A**) The RAG complex composed of RAG1 (purple) and RAG2 (green) binds to the 12- and 23RSSs (dark purple and orange triangles, respectively) neighboring gene segments (shown as red and yellow boxes on the DNA), (**B**) forming the paired complex (PC). At any point when it is bound to an RSS, RAG can introduce a nick in the DNA between the heptamer and gene segment (shown with the magnified 12RSS) and must do so to both sites before (**C**) it cleaves the DNA to expose the gene segments. As indicated by the magnified gene segment end, the exposed DNA strands of the gene segment are connected to form a DNA hairpin. (**D**) Additional proteins join these segments together. In this work, the stages subsequent to DNA cleavage are not monitored.

The process of V(D)J recombination (schematized in Figure 1) is initiated with the interaction between the recombination-activating gene (RAG) protein complex and two short sequences of DNA neighboring the gene segments, one that is 28 bp and another that is 39 bp in length. These recombination signal sequences (RSSs) are composed of a well-conserved heptamer region immediately adjacent to the gene segment, a more variable 12- (for the 12RSS) or 23-bp (for the 23RSS) spacer sequence and a well-conserved nonamer region. For gene rearrangement to begin, RAG must bind to both the 12- and the 23RSS to form the paired complex (PC) state (Figure 1B). Throughout the binding interaction between RAG and either RSS, RAG has an opportunity to nick the DNA (enlargement in Figure 1B) (6). RAG must nick both RSSs before it cleaves the DNA adjacent to the heptamers to expose the gene segments and to create DNA hairpin ends (Figure 1C). DNA repair proteins complete the reaction by joining the gene segments to each other and the RSSs to one another (Figure 1D).

RSS sequence-conservation studies across many organisms have shown a vast diversity of 12- and 23RSS sequences, mainly found through heterogeneity in the spacer region (7). Bulk assays reveal that changing an RSS sequence can significantly influence the RAG–RSS interaction and ultimately the success rate of completing recombination (8–12). Recent structural results provide evidence that RAG binding is sensitive to both base-specific contacts and the local flexibility or rigidity of the 12- and 23RSS (13–15). Despite this extensive characterization on the interaction, little is known about how a given RSS sequence affects each step of the RAG–RSS reaction. In this work, we provide one of the most comprehensive studies of how RSS sequences govern the initial steps of V(D)J recombination and provide a quantitative measure of their effects on the formation frequency, lifetime and cleavage probability of the PC.

**Figure 2.**
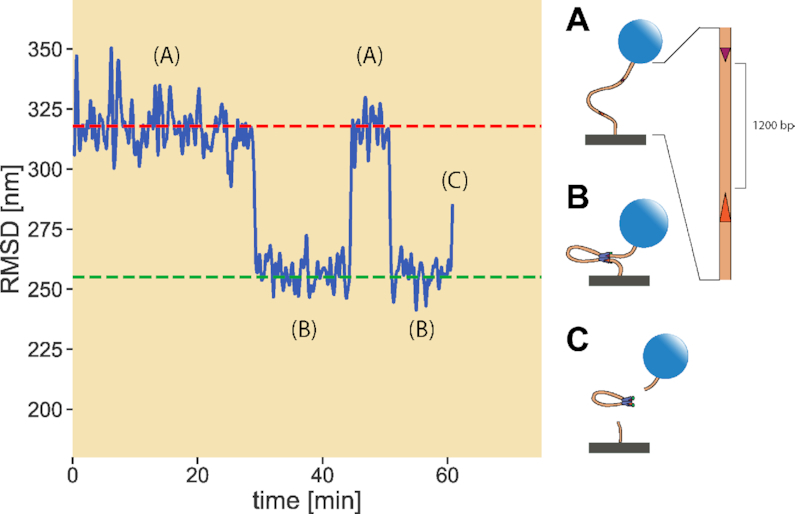
Sample data output of TPM. By tracking the root-mean square displacement (RMSD) of the tethered bead position undergoing restrained Brownian motion, we discern when the DNA tether is (**A**) in the unlooped state, (**B**) in the PC (looped) state and (**C**) cleaved. The dashed horizontal lines distinguish the unlooped (red) and looped (green) states of the DNA, and are drawn before examining the bead trajectories. The RMSD values of these lines are based on the length of the DNA tether; the distance between the RSSs along the strand; the extent to which HMGB1, a protein that binds nonspecifically to DNA and helps facilitate RAG binding, kinks the DNA; and a set of calibration experiments relating the range of motion of the bead to the length of its tether. As depicted with the magnified DNA strand in (A), the 12RSS and 23RSS are positioned 1200 bp away throughout the study.

We employ a single-molecule technique known as tethered particle motion (TPM) in which an engineered strand of DNA containing a 12RSS and 23RSS is attached to a glass coverslip at one end and to a polystyrene bead at the other (Figure 2A). Using brightfield microscopy, we collect the root-mean-squared displacement (RMSD) of the bead over time to identify the state of the RAG–RSS interaction. As illustrated in Figure 2B, when RAG forms the PC with the RSSs, the shortened DNA tether constrains the motion of the bead, reducing the RMSD. When RAG cleaves the PC, the bead is released and diffuses away from the tether site (Figure 2C). TPM has been applied to track the dynamic behavior of various protein–DNA systems, including RAG and RSS (16–21). It is with the temporal resolution provided by TPM that we can track the full progression of individual RAG–RSS interactions from PC formation to cleavage.

We were interested in using TPM to determine the extent to which endogenous RSSs dictate the usage frequency of their neighboring gene segments and, for those RSS positions that do seem to influence gene segment usage, identify the steps in the RAG–RSS reaction when the RSSs help or hurt the selection of their gene segment by extracting kinetic rates. We first examine single bp changes to a designated reference 12RSS, thereby establishing a mechanistic understanding of the contribution of individual nucleotide positions to RAG–RSS dynamics. With the synthetic 12RSSs providing context, we study a set of endogenous 12RSSs, each of whose sequences can be directly related to the reference sequence and a subset of the characterized synthetic 12RSSs. This selection of 12RSSs was also chosen from repertoires where the usage frequencies of their gene segments are known. Finally, due to the depth of insight offered by waiting time distributions generated by the TPM assay, in an attempt to provide some of the first measurements of various RAG–RSS kinetics, we show through our analysis of the PC lifetime distributions that regardless of choice of 12RSS or divalent cation, our TPM data consistently disagree with a single-rate model. We discuss the consequences of our finding in the context of our understanding of the molecular details of the RAG–RSS reaction. As this study resulted in a wealth of data on a large number of RSS sequences, we have developed an interactive online resource for visualizing the dataset in its entirety.

## MATERIALS AND METHODS

### Protein purification

The two RAG components, core RAG1 and core RAG2 (RAG1/2), are purified together as outlined in Ref. (20). Maltose binding protein-tagged murine core RAG1/core RAG2 were co-expressed by transfection in HEK293-6E suspension cells in a 9:11 w/w ratio for 48 h before purifying using amylose resin. HMGB1 is purified as outlined in (20). His-tagged HMGB1 was expressed in isopropyl β-d-1-thiogalactopyranoside-induced BL21 cells for 4 h at 30°C before purification. For more details, see the Supplementary Data Text.

### Flow cell assembly

TPM flow cells were assembled by drilling four holes along each length of a glass slide before cleaning the slides and coverslips. The slides and coverslips were functionalized with an epoxidizing solution for at least an hour and a half so that anti-digoxigenin, to which the digoxigenin ends of the DNA tethers attach, could adhere to the glass. Upon completion of the treatment, flow cells are assembled by cutting four channels into double-sided tape to connect the drilled holes at opposite ends of the glass slide before adhering to the coverslip on one side and the glass slide on the other. Short connective tubes are inserted into each of the holes to serve as inputs and outputs for fluids and sealed using 5-min epoxidizing solution. The constructed flow cells are baked on the hot plate to allow the epoxy and double-sided tape to set.

### Tethered bead assembly

Tethered beads are assembled as in Supplementary Figure S1. Flow cell channels are incubated with anti-digoxigenin for 2 h to allow for adhering DNA to the glass surfaces. After washing away excess anti-digoxigenin in a buffer solution containing Tris-HCl, KCl, MgCl_2_, DTT, EDTA, acetylated BSA and casein, engineered strands of 2900 bp-long DNA containing a 12RSS and a 23RSS located 1200 bp apart and tagged with digoxigenin on one end and biotin at the other end are injected into the flow cells to attach the digoxigenin end of the DNA to the anti-digoxigenin-scattered surfaces. After excess DNA is washed out, streptavidin-coated polystyrene beads 490 nm in diameter are added to the channels and incubated for ≈3 min to bind the biotin-labeled end of the DNA. Excess beads are washed away and the TPM assembly buffer is replaced with a RAG reaction buffer containing Tris-HCl, KCl, glyercol, DTT, potassium acetate, MgCl_2_, DMSO and acetylated BSA. For Ca^2 +^ studies, CaCl_2_ is used in place of MgCl_2_ in the RAG reaction buffer and in the same concentration. See Supplementary Data Text for a schematic of the TPM assembly process.

### TPM experiment

TPM experiments involve the simultaneous acquisition of bead trajectories from two different channels on separate microscopes. One of the channels contains tethered DNA with a 12RSS and a 23RSS oriented toward each other (nonamer regions on both RSSs closest to each other). Properly tethered beads are filtered using various methods to ensure proper spacing from neighboring beads and that individual beads are tethered by a single strand of DNA. The trajectories of the selected beads are then examined in the absence of RAG and HMGB1 for 10 min before flowing in 9.6 nM murine core RAG1/core RAG2 and 80 nM full-length HMGB1 and acquiring bead trajectories for at least 1 h. Root-mean-squared displacements (RMSDs) of the bead trajectories as shown in Figure 2 are calculated by Gaussian filtering with an 8-s standard deviation. Bead selection criteria, corrections and smoothing of trajectories, and identification of PCs are provided in the Supplementary Data Text. Example dataset of all analyzed bead trajectories from one replicate is presented in Supplementary Figure S2.

### Statistical inference

We used Bayesian and Frequentist methods in this work to calculate parametric and nonparametric quantities, respectively. The PC formation frequencies were assigned confidence intervals via bootstrapping. Briefly, the observed beads and their reported PC formation counts were sampled with replacement to generate a simulated data set of the same length as the number of observations. The looping frequency was then calculated as the total loops formed among the generated dataset divided by the number of beads and the distribution was resampled again. This procedure was performed 10^6^ times and we report various percentiles of these bootstrap replicates, as shown both in the main text and on the paper website. A more detailed explanation is provided in the Supplementary Data Text.

To compute the cleavage probability and PC leaving rate *k*_leave_, we used a Bayesian definition of probability and constructed a posterior distribution for each as is explicitly laid out in the Supplementary Data Text. The displayed posterior distributions for the cleavage probability were generated by numerically evaluating the posterior distribution over a range of cleavage probabilities bounded from 0 to 1. The reported values for the cleavage probability and uncertainty were computed analytically and is derived in the Supplementary Data Text.

To estimate *k*_leave_ we again constructed a posterior distribution. Here, we chose an exponential form for the likelihood and assumed an inverse Gamma distribution as a prior on the leaving rate. This posterior was then sampled using Markov chain Monte Carlo as is implemented in the Stan probabilistic programming language (22). A more detailed derivation of the posterior distribution is provided in the Supplementary Data Text. All models and code for this inference are available on the paper website.

Significance testing was performed for the looping frequency, median PC lifetime, and fraction of cutting events. Our null hypothesis for each metric was that the measured value for the altered 12RSS was drawn from the same distribution as the V4-57-1 (reference) 12RSS with *p*-values ≤ 0.05 determined to be statistically significant. All *p*-values for each of these metrics and details about their calculation are provided in the Supplementary Data Text.

### Interactive figures

All results presented in this manuscript are visually complemented with interactive figures on the paper website at https://www.rpgroup.caltech.edu/vdj_recombination/. The Cutting Probability Model Explorer shows how the posterior distribution for the cutting probability changes depending upon the number of loops and number of cuts observed, both of which can be adjusted with their respective scroll bars. The Synthetic RSS Explorer page displays data for synthetic RSSs. Clicking on individual cells in the paired complex formation frequency, paired complex dwell time or paired complex cleavage probability heatmaps reveals plots of the looping frequencies with different confidence interval percentages from 10^6^ bootstrap replicates; empirical cumulative distribution functions (ECDFs) of the PC lifetimes that revert to an unlooped configuration, are cut, or a combination of the two fates; and full posterior distributions of the probability of cutting for the synthetic RSS in blue and the reference RSS in gray. Number of beads, loops and cuts observed for the synthetic RSS are displayed by hovering over the cells of the heatmaps. The Endogenous RSS Explorer page displays these same plots but allows for comparison between any two endogenous RSSs studied through dropdown menus, with the data for one RSS displayed in gray, including observation counts, and those for the other RSS shown in blue. The Synthetic-Endogenous RSS Comparison tool provides a means for selecting a particular endogenous RSS by a dropdown menu and directly comparing data for the endogenous RSS (gray) and the individual synthetic RSS that constitutes the sequence difference between the endogenous RSS and the V4-57-1 (reference) RSS, as revealed in the endogenous sequence with highlighted letters where the endogenous and reference RSSs differ.

## RESULTS

### Synthetic RSSs

We chose a 12RSS flanking the immunoglobulin κ variable (*Igκ*V) gene segment, V4-57-1, as the reference sequence due to its use in a previous TPM study on RAG–RSS interactions (20). This sequence has also been used in structural studies of RAG–RSS complexes (13,15), allowing us to compare our results with known information on the RAG–RSS structure. To explore how RAG–RSS interactions are affected by single bp changes, we examined 40 synthetic RSSs consisting of single bp changes across 21 positions of the V4-57-1 12RSS, with a particular focus on altering the 12 bp spacer that is the least well-understood element in the RSS. We also studied changes made to positions 3–7 of the heptamer and various positions of the nonamer. The first three positions of the heptamer are perfectly conserved (7) likely to support DNA distortions needed for both nicking and base-specific interactions with the cleavage domain on RAG1 after nicking (13–15), while heptamer positions 4–7 also mediate base-specific interactions with RAG (13). The nonamer is bound by a nonamer-specific binding domain on RAG1 (13,23). Throughout our synthetic and endogenous RSS study, we used the same concentration of the two RAG components (RAG1 and RAG2) that were co-expressed and co-purified; and the same concentration of the high mobility group box 1 (HMGB1) protein, which binds nonspecifically to DNA and helps facilitate RAG binding to the RSSs (12). We also fixed the distance between the two binding sites to be 1200 bp, thereby constraining our study to the influence of binding site sequence on RAG–RSS dynamics alone. In addition, all of the 12RSSs in this study are partnered with a well-characterized 23RSS (13,15,20) adjacent to the frequently used Jκ1 gene segment from the mouse *Ig*κ locus on chromosome 6 (5). The sequence of this RSS is provided in Supplementary Table S1. All primer sequences and bead, loop and cut counts for each synthetic 12RSS are provided in Supplementary Table S2.

**Figure 3.**
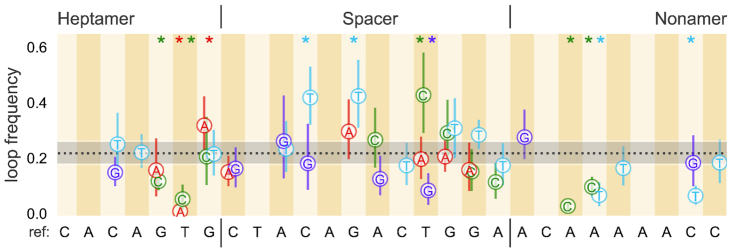
Looping frequency for single bp changes introduced at various positions of the reference 12RSS. Loop frequency with 95% confidence interval of the distribution of possible looping frequencies from 10^6^ bootstrap replicates. The dotted black line is set at the reference loop frequency, 0.22, with shaded area denoting the extent of the 95% confidence interval for the reference. Alternating vertical stripe colors and the reference sequence written along the *x*-axis demarcate the position where the change was made and the original nucleotide. The introduced nucleotide is provided in the figure with the letter and color-coded (red for A, green for C, light blue for T and purple for G). Heptamer, spacer and nonamer regions are also separated by vertical lines in the sequences. Asterisks at the top of certain positions are color-coded to specify the nucleotide whose resultant looping frequency differs from the reference sequence with *p*-value ≤ 0.05. All *p*-values for each 12RSS used are reported in Supplementary Figure S5.

We pooled the relevant data across experimental replicates to characterize synthetic RSSs by three empirical properties, namely the frequency of entering the PC (looping frequency), the quartiles of the PC lifetime (dwell time) distribution and the probability of exiting the PC through DNA cleavage (cutting probability). We define the looping frequency as the ratio of distinct PCs observed to the total number of beads monitored over the course of the experiment. Because a single DNA tether can loop and unloop multiple times over the course of the experiment, the looping frequency can in principle range from 0 to ∞. The measured looping frequency and the 95% confidence intervals from bootstrapping the looping frequency (as demonstrated in Supplementary Figure S3) are shown for all of the synthetic RSSs in Figure 3.

**Figure 4.**
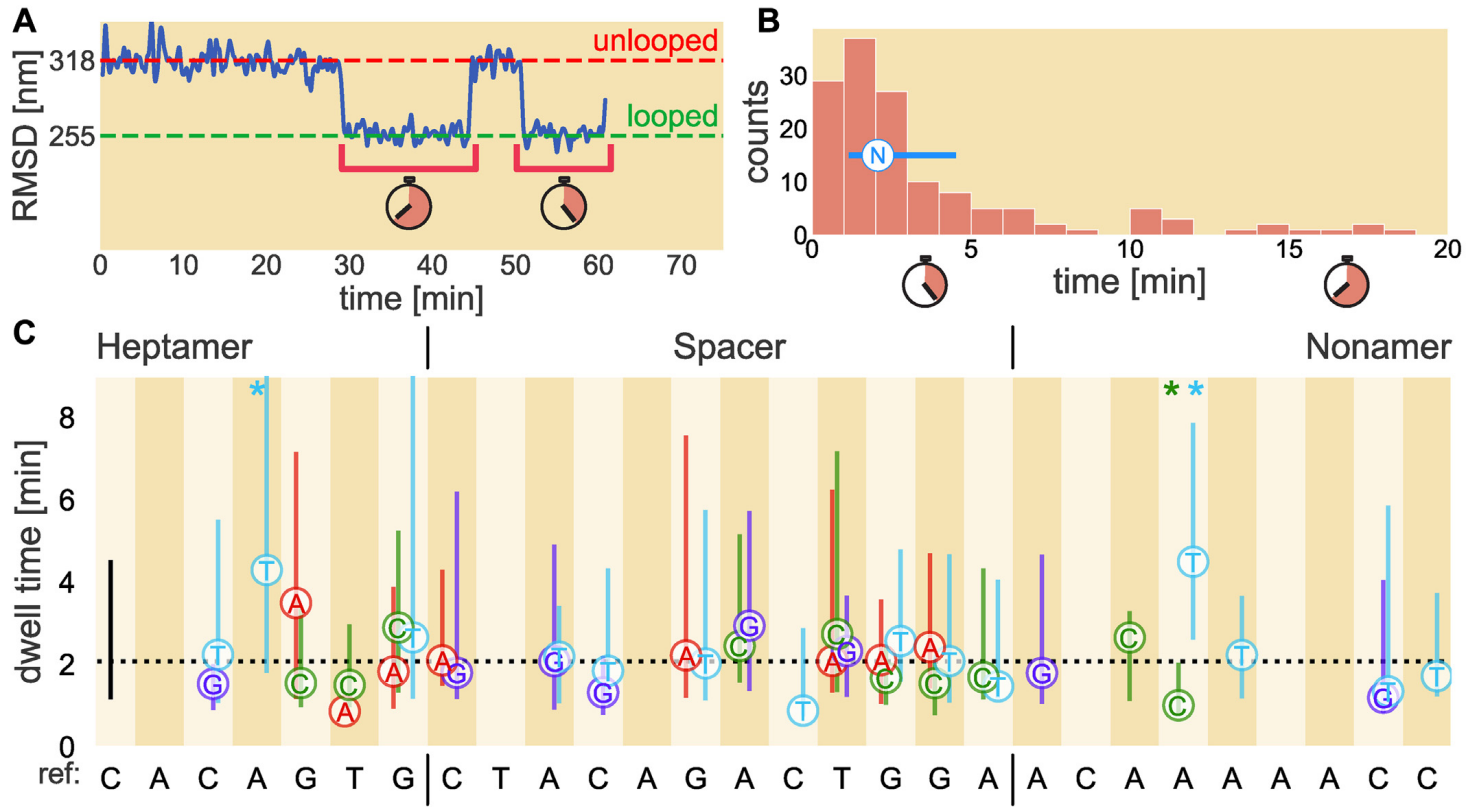
Dwell time quartiles for single bp changes introduced at various positions of the reference 12RSS. (**A**) Example bead trajectory data (blue) and the dwell times of the two loops that are formed (brackets). As in Figure 2, the red dashed line corresponds to the unlooped DNA tether state while the green dashed line denotes the predicted looped state. (**B**) Histogram of all dwell times collected for a given RSS. Note that all loops involving the RSS of interest are included in the histogram, regardless of whether the loop precedes cutting or a return to the unbound state. The median is shown as the circle containing N (for nucleotide) with lines extending to the first and third quartiles. The method for obtaining the circle and error bars as shown in (**B**) are then applied to each synthetic 12RSS dataset and presented in (**C**) where the letters denote the replacement nucleotide. The dotted black line in (C) denotes the reference 12RSS median dwell time, 2.1 min, with the black bar at the left denoting the first and third quartiles of the distribution. Vertical stripes; *x*-axis labeling; heptamer, spacer and nonamer distinction; and color-coding of nucleotide changes (red for A, green for C, light blue for T and purple for G) are the same as in Figure 3. Asterisks at the top of certain positions are color-coded to specify the nucleotide whose resultant dwell time differs from the reference sequence with *p*-value ≤ 0.05. All *p*-values for each 12RSS used are reported in Supplementary Figure S5.

As demonstrated in Figure 4A, the dwell times were obtained from measuring the lifetimes of each PC state, irrespective of whether the PC was cleaved or reverted to an unlooped state. For each synthetic RSS, all of the PC lifetimes are pooled to generate a histogram of dwell time distributions such as that in Figure 4B, from which the mean, shown as a white circle with an N for nucleotide, and the first and third quartiles, shown as the furthest extents of the blue error bar, are used to compare the synthetic RSSs in Figure 4C.

**Figure 5.**
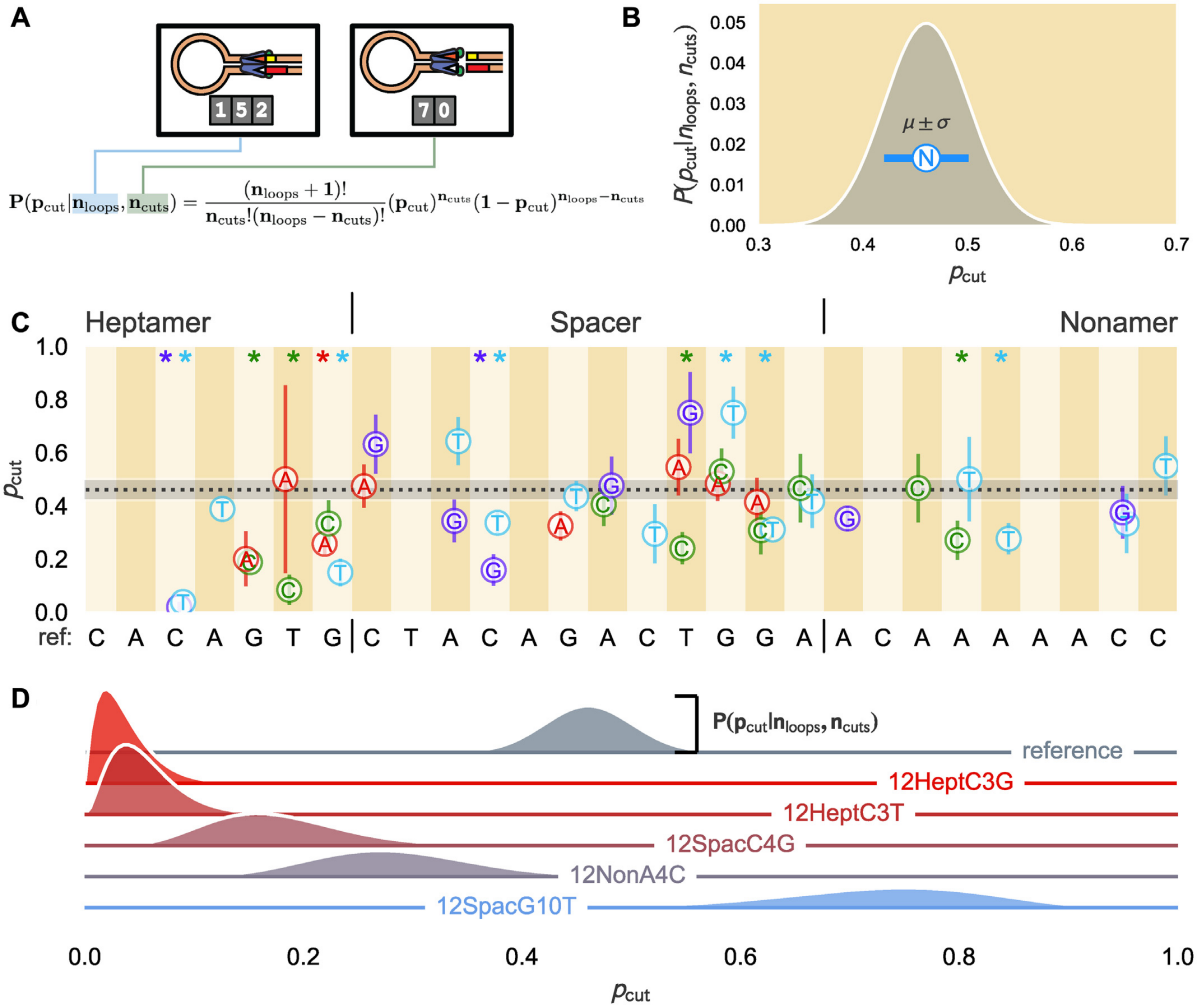
Cutting probabilities for single bp changes introduced at various positions of the reference 12RSS. (**A**) For a given RSS, the total number of distinct loops in the assay *n*_loops_ (in this case, 152 loops) and the subset of those loops that RAG cleaves *n*_cuts_ (70) are applied to the function shown that identifies the full distribution of the cutting probability *p*_cut_ of the PC for a 12RSS of interest. (**B**) Example distribution for a particular RSS, with the most likely cutting probability *μ* with N for ’nucleotide’ and standard deviation *σ* shown as a circle with blue error bars, respectively. (**C**) *μ* and *σ* are shown for each synthetic RSS with the dotted black line denoting the most probable *p*_cut_ for the reference sequence, roughly 0.46, with the gray shaded region setting one standard deviation. Vertical stripes; *x*-axis labeling; heptamer, spacer and nonamer distinction; and color-coding of nucleotide changes (red for A, green for C, light blue for T and purple for G) are the same as in Figure 3. (**D**) Ridgeline plot of posterior distributions of the cutting probability, given the number of loops observed and loops that cut (see Supplementary Data) for a subset of the synthetic RSSs (labeled and colored along the zero-line of the respective ridgeline plot). Height of the distribution to the horizontal line of the same color corresponds to the posterior distribution. See the Cutting Probability Model Explorer interactive webpage to see how the posterior distribution depends on the number of loops and cuts observed. Asterisks at the top of certain positions are color-coded to specify the nucleotide whose resultant cutting probability differs from the reference sequence with *p*-value ≤ 0.05. All *p*-values for each 12RSS used are reported in Supplementary Figure S5.

Finally, to compute the cutting probability, we considered the fate of each PC as a Bernoulli trial with cleavage probability *p*_cut_. This treatment allows us to construct the full probability distribution of *p*_cut_ defined explicitly in Figure 5A and fully detailed in the ‘Materials and Methods’ section and Supplementary Data Text for a PC containing the RSS of interest. The measured number of loops *n*_loops_ and cuts *n*_cuts_ collected from experiments (in the case of Figure 5A, 152 loops and 70 cuts) are parameters inserted into the equation to yield a distribution such as in Figure 5B. We computed the most likely *p*_cut_ and one standard deviation, as demonstrated in Figure 5B by the white circle with the N and blue error bars, respectively, for each synthetic RSS, and compiled them in Figure 5C. The Cutting Probability Model Explorer interactive figure provides a visualization for how the probability distribution is sensitive to the empirically-collected number of loops and cuts. Detailed discussions of the choice of metrics and the corresponding error estimates are provided in ‘Materials and Methods’ section and Supplementary Data Text. We also show in Supplementary Figure S4 and Supplementary Data Text that our definitions of the looping frequency and cutting probability decouple the PC forming and cleavage steps in the RAG–RSS reaction, thereby clarifying which step is the limiting factor in completing the cleavage phase of V(D)J recombination. We complement the condensed synthetic RSS results presented here with an interactive figure that provides a more complete visualization of each RSS studied on the website. The Synthetic RSS Explorer interactive figure includes heatmaps to qualitatively illustrate how the synthetic RSSs differ in the three defined metrics. By clicking on a particular cell in any of the heatmaps, the interactive displays the measured looping frequency of the synthetic RSS containing the corresponding bp change with several confidence interval percentages from the bootstrapping. Hovering over a cell also brings up a window showing the number of beads, loops and cuts observed for the synthetic RSS. In addition, the webpage shows empirical cumulative distribution functions (ECDFs) of PC lifetimes in three groups: PCs that are cleaved, PCs that are unlooped and both together. This webpage includes the complete posterior probability distribution of *p*_cut_ for each synthetic RSS.

Figures 3, 4C and 5C illustrate the substantial effect that a single bp change to an RSS can have on the formation, stability and cleavage of the PC, respectively, reaffirming that RSS sequence plays a role in regulating the initial steps of recombination. Of interest is the observed difference in phenomena between changes made to the third position and those made to the last four bases of the heptamer region. Bulk assays have shown that deviating from the consensus C at heptamer position 3 essentially eliminates recombination (8,10), yet we found that changing from the C to G or T did not inhibit PC formation (Figure 4C). In fact, these alterations show similar looping frequencies and PC lifetimes (Figure 4C) as found for the reference sequence. Instead, both the C-to-G and C-to-T alterations to heptamer position 3 almost completely suppress cleavage (Figure 5C). We provide the full probability distribution for the estimate of *p*_cut_ for these two RSSs in Figure 5D. Nearly all of the probability density is concentrated below 10%, showing that cutting the PC is exceedingly rare. Thus, although deviating from a C at heptamer position 3 does not prevent RAG from forming the PC, the alteration impedes DNA cleavage.

Among the changes made to the last four bases of the heptamer from the reference sequence, the fifth and sixth positions showed the most striking reductions on PC formation (Figure 3). Of >240 DNA tethers with the 12RSS containing a T-to-A change at heptamer position 6, only two PCs formed, one of which subsequently led to cleavage. This result is consistent with recent findings that the consensus TG dinucleotide at the last two positions of the heptamer supports a kink in the DNA and may be critical for RAG binding (14). We notice that some changes such as the one at heptamer position 4 (A to T) increase the median time spent in the PC (Figure 4C). This RSS also had one of the widest dwell time distributions of all of the synthetic RSSs studied. While some alterations to the last four heptamer positions yielded little change in cleavage propensity compared to the reference, others showed a reduction in *p*_cut_. The single bp change that had the greatest effect, located at heptamer position 6 (T to C) showed that only 2 out of 24 PCs led to cleavage.

Although we observed only modest differences in the median dwell times when we altered the reference sequence in the spacer region, some alterations substantially affected the looping frequency and cutting probability. The C-to-T change at spacer position 4 doubled the frequency of observing the PC while a T-to-G change at the ninth position reduced PC formation nearly as much as changes made at heptamer position 6 (Figure 3). These two changes made in the spacer reflect the observed extremes of spacer sequence effects on the looping frequency. While many of the changes in the spacer region do not alter the cutting probability, we can still find spacer-altered RSSs that improve or inhibit cleavage. Figure 5D shows that changing the fourth position from C to G shifts the probability distribution of *p*_cut_ to lower values, while altering the tenth position of the spacer from G to T shifts the distribution toward an increased cleavage probability. RAG1 makes contacts along the entire length of the 12RSS spacer (14), helping to explain our finding that changes to the spacer can substantially alter the probability of PC formation and cutting, thereby playing more of a role than simply separating the heptamer and nonamer sequences.

Similar to spacer changes, most nonamer changes show strongly overlapping dwell time distributions, with median PC dwell times differing from the reference sequence by <1 min (Figure 4C). However, unlike spacer-modified RSSs, most nonamer-altered RSSs reduced the frequency of PC formation. Disruptions to the poly-A sequence in the center of the nonamer cause a substantial reduction in looping frequency, most notably the near complete inhibition of PC formation with the A-to-C change at nonamer position 3 (Figure 3). This detrimental effect of deviating from the poly-A tract agrees with previous work demonstrating numerous protein–DNA interactions in this region and with the proposal that the rigidity produced from the string of A nucleotides is a critical feature for RAG1 to bind the nonamer (14,23). Furthermore, this reduction in looping frequency can extend to changes made toward the end of the nonamer, depending upon the nucleotide, as shown with the significant reduction for the C-to-T mutation at nonamer position 8 (Figure 3). The sequence deviations in the nonamer region, however, do not significantly affect cleavage once the PC has formed, as evidenced by the overlap in the posterior distributions of the reference sequence and its nonamer variant showing the greatest reduction in cleavage probability (position 4, A to C), in Figure 5D. Overall, nonamer deviations from the reference RSS have negative effects on PC formation with minimal effects on subsequent DNA cleavage, consistent with extensive biochemical and structural evidence that the primary function of the nonamer is to facilitate RAG–DNA binding (23).

### Endogenous RSSs

To build on our study of single bp effects on RAG–RSS dynamics, we selected a subset of endogenous RSSs from the mouse Vκ locus on chromosome 6 based on existing gene usage frequency data collected by Aoki-Ota *et al.* (5) and because the sequence differences between these RSSs and the reference RSS are individually examined in the synthetic RSS results. We studied a variety of frequently used (>5% frequency of usage) gene segments (V1-135, V9-120, V10-96, V19-93, V6-15 and V6-17), two moderately used (>1% and <3% frequency) gene segments (V4-55 and V5-43) and two rarely used (<0.5% frequency) gene segments (V4-57-1 and V8-18) (5). We note that the V4-57-1 12RSS is identical to the reference 12RSS in the synthetic study. Furthermore, we use the same Jκ1 23RSS in the endogenous RSS study as in the synthetic study. In addition, we examined DFL16.1, the most frequently used D gene segment from the murine immunoglobulin heavy chain (*Igh*) locus on chromosome 12 (4,24). Unlike the Vκ gene segments, which only need to combine with one gene segment, D gene segments must combine with two other gene segments to encode a complete protein. As a result, DFL16.1 is flanked on both its 5′ and 3′ sides by distinct 12RSS sequences, denoted DFL16.1-5′ and DFL16.1-3′, respectively, both of which are examined in this study. The sequences of all endogenous RSSs studied here as well as the number of beads, loops and cuts observed are provided in Supplementary Tables S1 and S3. We apply TPM on these sequences to determine whether their involvement in the RAG–RSS reaction could both provide insight into the usage frequency of their flanking gene segments and be predicted based on the activity profile of the synthetic RSSs.

**Figure 6.**
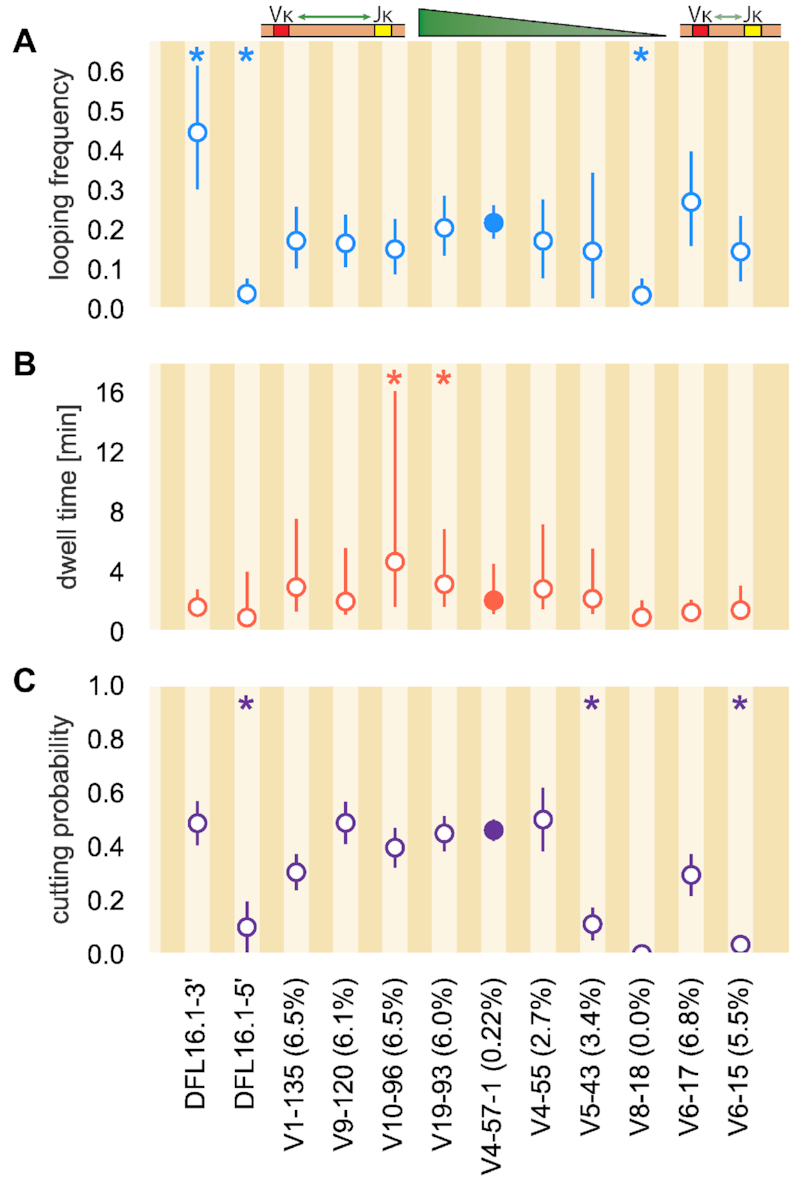
Observed dynamics between RAG and endogenous RSS sequences. (**A**) Frequency of PC formation (looping frequency) with 95% confidence interval. (**B**) Median PC lifetime with the lower error bar extending to the first quartile and the upper error bar extending to the third quartile. (**C**) Probability of DNA cleavage (cutting probability) of RAG with error bars showing one standard deviation. For discussion of the errors in Figure 6A and C, see the Supplementary Data Text. DFL16.1-3′ and DFL16.1-5′ flank the same gene segment but in different orientations on the Igh chromosome. As shown in the graphic above Figure 6A, Vκ gene segments listed are ordered by their position along the chromosome, with linear distance from the Jκ gene segments decreasing from left to right. Numbers in parentheses next to Vκ gene segment denote percentage of usage in repertoire (5). The V4-57-1 12RSS has a filled in circle to denote that it is the reference sequence in the synthetic RSS study. Asterisks at the top of subfigures denote endogenous RSSs whose measured quantity differs from the V4-57-1 (reference) 12RSS with *p*-value ≤ 0.05. All *p*-values for each 12RSS used are reported in Supplementary Figure S5.

To develop a better sense for how RAG interacts with these RSSs in their endogenous context, the 6 bp coding flank sequence adjacent to the heptamer of all but the V4-57-1 RSS was chosen to be the natural flank provided by the endogenous gene segment. RAG interacts with the coding flank during DNA binding and PC formation (13–15) and coding flank sequence can influence recombination efficiency, particularly the two bp immediately adjacent to the heptamer (25–27). Two T nucleotides and in many cases even a single T immediately 5′ of the heptamer inhibit the nicking step of cleavage and thus reduce recombination efficiency (25–27). We did not extensively analyze the contribution of coding flank sequence in this study, and only V6-15 RSS among the studied RSSs would be predicted to interact poorly with RAG due to the T flanking the heptamer; all other coding flanks have combinations of A and C as the two terminal coding flank bases. We kept the same coding flank for the V4-57-1 RSS as in a previous study (20) to facilitate closer comparison of the results of the synthetic RSSs. We do not expect much difference between the endogenous coding flank sequence (5′-CACTCA, where the two nucleotides closest to the heptamer are underlined) and the coding flank used here (5′-GTCGAC) because the two terminal coding flank bases are similar to those of all but the V6-15 RSS and for reasons discussed in the ‘Discussion’ section and Supplementary Data Text. The coding flank sequences for all studied endogenous RSSs are included in Supplementary Table S1. We present the results of the RAG-endogenous RSS interaction in Figure 6 and provide an interactive tool for exploring these data on the paper website. The Endogenous RSS Explorer includes an interactive feature where the looping frequency, ECDFs of looping lifetimes, and posterior distributions of the cleavage probability of any two endogenous RSSs can be directly compared.

The variable nature of all three metrics [looping frequency (Figure 6A), dwell time (B) and cutting probability (C; full posterior distributions for all endogenous 12RSSs studied here are shown in Supplementary Figure S6) across RSSs highlights how, similar to the synthetic RSSs, endogenous sequences influence formation, stability and cleavage of the PC differently. Of particular interest is the behavior of DFL16.1-3′ that shows the highest propensity for PC formation but some of the shortest PC lifetimes. Despite this short median dwell time, the probability of the PC successfully proceeding to DNA cleavage is high, ≈0.5. Notably, the frequency of PC formation and the probability of cleavage are both greatly reduced for DFL16.1-5′ as compared to DFL16.1-3′, although their median PC dwell times and the width of the dwell time distributions are approximately equal. Reduced function of DFL16.1-5′ relative to DFL16.1-3′ is consistent with prior studies (24,28,29) and is addressed further in the ‘Discussion’ section.

The endogenous RSSs of the Vκ gene segments show varying efficiencies of PC formation and cleavage. Many of the endogenous RSSs studied here, including those of gene segments used frequently *in vivo* (V1-135, V9-120, V10-96, V19-93, V6-17 and V6-15) demonstrate looping frequencies between 15 and 30 events per 100 beads. Gene segments V4-57-1 and V4-55 are used with almost 0% and roughly 2.5% frequency, respectively (5), yet in our experiments, they enter the PC with comparable frequency (∼20–30 loops per 100 beads). In general, we find these two sequences to behave almost identically in our experimental system, illustrating that other biological phenomena, such as higher order DNA structure, govern the segment usage *in vivo* (4,30). The endogenous V8-18 12RSS exhibits infrequent PC formation and cleavage and short median PC lifetimes, much like the DFL16.1-5 12RSS. Using the V8-18 12RSS, only 5 looping events were detected from 146 DNA tethers and cleavage was never observed. Despite the similarities in reaction parameters for the V8-18 and DFL16.1-5′ RSSs, DFL16.1 is the most frequently used D gene segment in the repertoire (4) while V8-18 is never used (5). A likely explanation for the exclusion of V8-18 in the repertoire is the ‘A’ at heptamer position 6 of the 12RSS (see ‘Discussion’ section). In contrast, the DFL16.1 is substantially utilized in the *Igh* repertoire despite the poor contribution in PC formation and cleavage of its 5′ 12RSS most likely because this RSS does not participate in recombination until after its gene segment has undergone D-to-J recombination with its more efficient 3′ 12RSS, thus moving the gene segment into the RAG-rich environment of the ‘recombination center.’ This relocation is thought to facilitate RAG binding to the 5′ RSS of the committed D gene segment (31,32).

Figure 6B demonstrates that, with the exception of the V10-96 RSS, PC lifetimes are similarly distributed across the endogenous RSSs examined in this work. Most RSSs have median dwell times between 1 and 3 min with the V8-18 12RSS displaying the shortest lived median dwell time of roughly 40–50 s. While most endogenous RSSs here have a similar range between the first and third quartiles (see Endogenous RSS Explorer interactive figure on the paper website), the V10-96 12RSS distribution is noticeably wider, with the first quartile of the distribution being a longer lifetime than the median lifetime for most endogenous RSS distributions and the third quartile of this RSS extending out to over 16 min. These observations suggest a similar stability of the PC for all but the V10-96 RSS once RAG manages to bind simultaneously to both 12- and 23RSSs.

Figure 6C indicates that six endogenous RSS sequences from V1-135 to V4-55 have comparable cutting probabilities ranging from 0.4 to 0.5. Considering that the less-frequently used V4-57-1 and V4-55 gene segments have 12RSSs that show similar cutting probabilities and looping frequencies to the 12RSSs of more frequently selected gene segments, other factors appear to prevent their efficient use. The low probability of cutting (0.05; Figure 6C) with the V6-15 12RSS is particularly noteworthy, indicating that RAG tends to easily break the looped state rather than commit to cleavage. However, this low cutting probability might be attributed to the T in the coding flank immediately adjacent to the heptamer. Other features of the system must dictate the high-frequency usage of V6-15 *in vivo* (5).

### Kinetic modeling of the PC lifetime distribution

Figures 4C and 6B show that the vast majority of median looping lifetimes ranged between 1 and 3 min with rare exceptions, suggesting similar dwell time distributions for many of the RSS variants. However, many of these synthetic and endogenous RSSs have different probabilities of DNA cleavage, suggesting that at the very least the rate of cutting changes. These similarities in the lifetime distributions but differences in outcomes invited a thorough dissection of the data to extract key quantitative insights into the changes in the kinetics between 12RSS constructs. As TPM has been used to extract kinetic parameters for various other protein–DNA systems (17,18,33,34), we used the distributions of the PC lifetimes in an attempt to establish the rates of unlooping and cutting for each RSS and discern a deeper connection between RSS sequence and fate of the PC. We developed a simple model in which a PC state can have two possible fates: either simple unlooping of the DNA tether or cleavage of the DNA by RAG. We characterized each of these outcomes as independent yet competing processes with rates *k*_unloop_ and *k*_cut_ for unlooping and DNA cleavage, respectively. If the waiting time distribution *t*_unloop_ or *t*_cut_ for each process could be measured independently where only one of the two outcomes was permitted to occur, one would expect the probability densities of these waiting times given the appropriate rate to be single exponential distributions of the form

(1) $$\begin{equation*} P(t_\mathrm{unloop}\, \vert \, k_\mathrm{unloop}) = k_\mathrm{unloop}e^{- k_\mathrm{unloop} \, t_\mathrm{unloop}} \end{equation*}$$

for the unlooping process and

(2) $$\begin{equation*} P(t_\mathrm{cut}\, \vert \, k_\mathrm{cut}) = k_\mathrm{cut}e^{-k_\mathrm{cut} \, t_\mathrm{cut}} \end{equation*}$$

for DNA cleavage. However, as these two Poisson processes are competing, we cannot estimate *k*_cut_ solely from the waiting time distribution of paired complex states that led to DNA cleavage nor *k*_unloop_ using the states that simply unlooped. As each individual cutting or unlooping event is assumed to be independent of all other cutting and unlooping events, the distribution of the dwell time *t* before the PC either unloops or undergoes cleavage can be modeled as an exponential distribution parameterized by the sum of the two rates,

(3) $$\begin{equation*} P(t\, \vert \, k_\mathrm{leave}) = k_\mathrm{leave} \, e^{- k_\mathrm{leave} \, t}, \end{equation*}$$

where *k*_leave_ = *k*_unloop_ + *k*_cut_.

**Figure 7.**
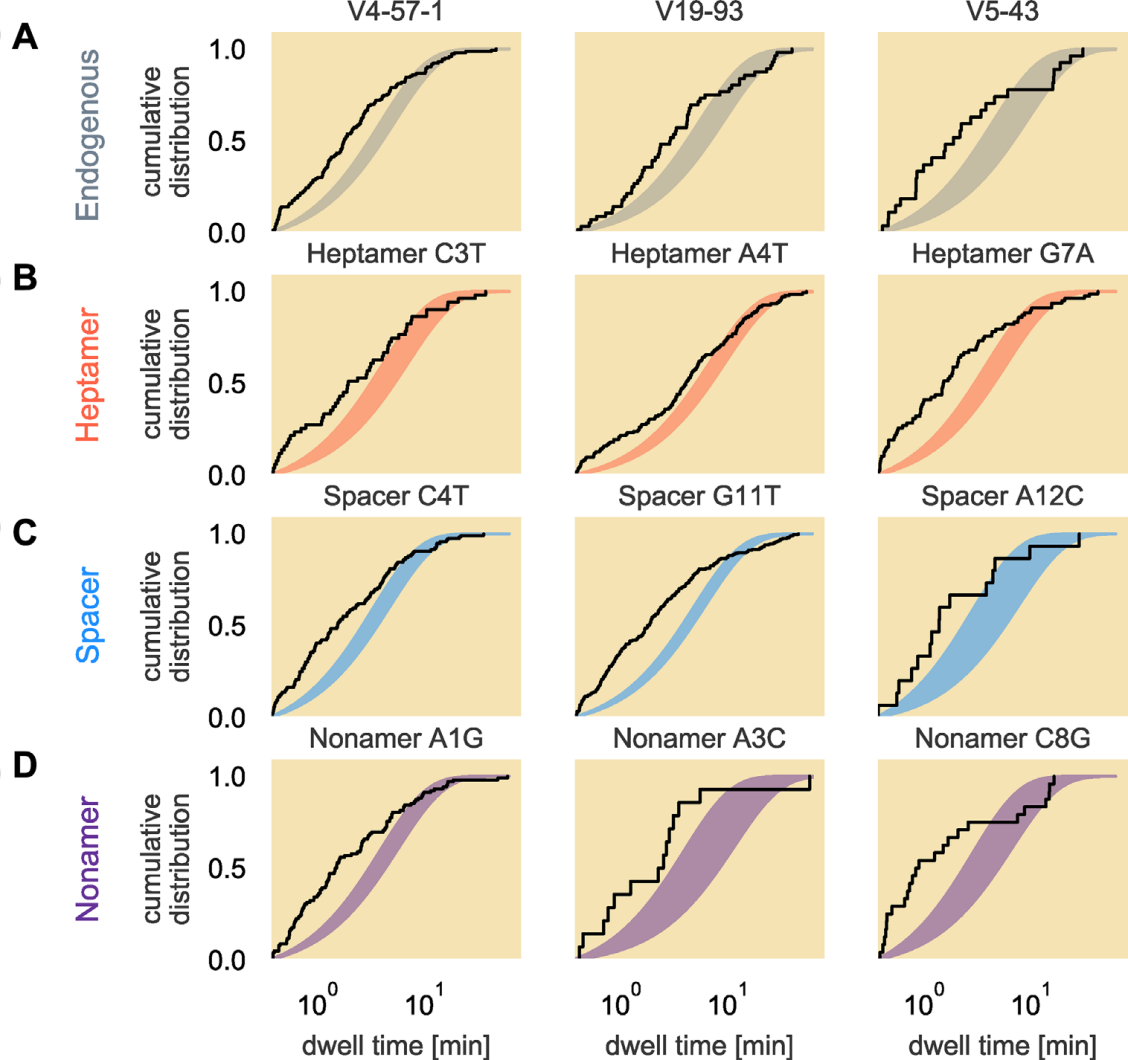
Non-exponential waiting time distributions for endogenous and synthetic 12RSSs. The empirical cumulative distribution of the measured PC lifetimes (black lines) are shown for representative endogenous sequences (**A**) as well as for the synthetic RSSs with single point alterations made in the heptamer (**B**), spacer (**C**) or nonamer (**D**) regions. The shaded area corresponds to the 95% credible region of a true exponential distribution parameterized in Equation (3) given a posterior distribution for *k*_leave_, the rate of the arrival of either an unlooping or cleavage event. All dwell time axes are plotted on a logarithmic scale.

Given the collection of waiting time distributions measured for each RSS, we estimated the values of *k*_leave_ that best describe the data. We find that the observed dwell times are not exponentially distributed for any 12RSS sequence analyzed, either endogenous or synthetic. Examples of these waiting time distributions along with an exponential distribution parameterized by the 95% credible region for *k*_leave_ can be seen for twelve of the RSS variants in Figure 7. In general, the observed dwell times are underdispersed relative to a simple exponential distribution with an overabundance of short-lived PCs. We also find that the observed dwell time distributions are heavily tailed with exceptionally long dwell times occurring more frequently than expected for an exponential distribution.

The ubiquity of this disagreement between the simplest kinetic model and the observed data across all of the examined RSSs indicates that leaving the PC state either by reverting to the unlooped state or committing to the cleaved state is not a one-step process, suggesting that at least one of the two fates for the PC state on its own is not single-exponentially distributed as assumed in our null model of the dynamics.

One hypothesis for the disagreement between the model given in Equation (3) and the data is that other processes, such as nicking of the DNA by RAG, create effects in the tethered bead trajectories that are too subtle to be detected in the TPM assays. Nicking creates a more stable RAG–single RSS complex (though this effect on PC stability had not been previously quantified) (13,35) and can occur at any time after RAG binds to the RSS (6), making it exceedingly difficult to determine whether a given PC has one, both or neither of the RSSs nicked. As a result, we may not be able to model the combined kinetics of unlooping and cleavage without also identifying when RAG nicks the RSSs to which it is bound.

Substitution of Ca^2+^ in place of Mg^2+^ in the reaction buffer allows RAG to bind the RSSs but blocks both nicking and cleavage (36), leaving unlooping as the only possible fate of a PC. To determine if unlooping could be modeled as a simple Poisson process, we measured the PC dwell time distribution for a subset of the RSSs in a reaction buffer containing Ca^2+^.

**Figure 8.**
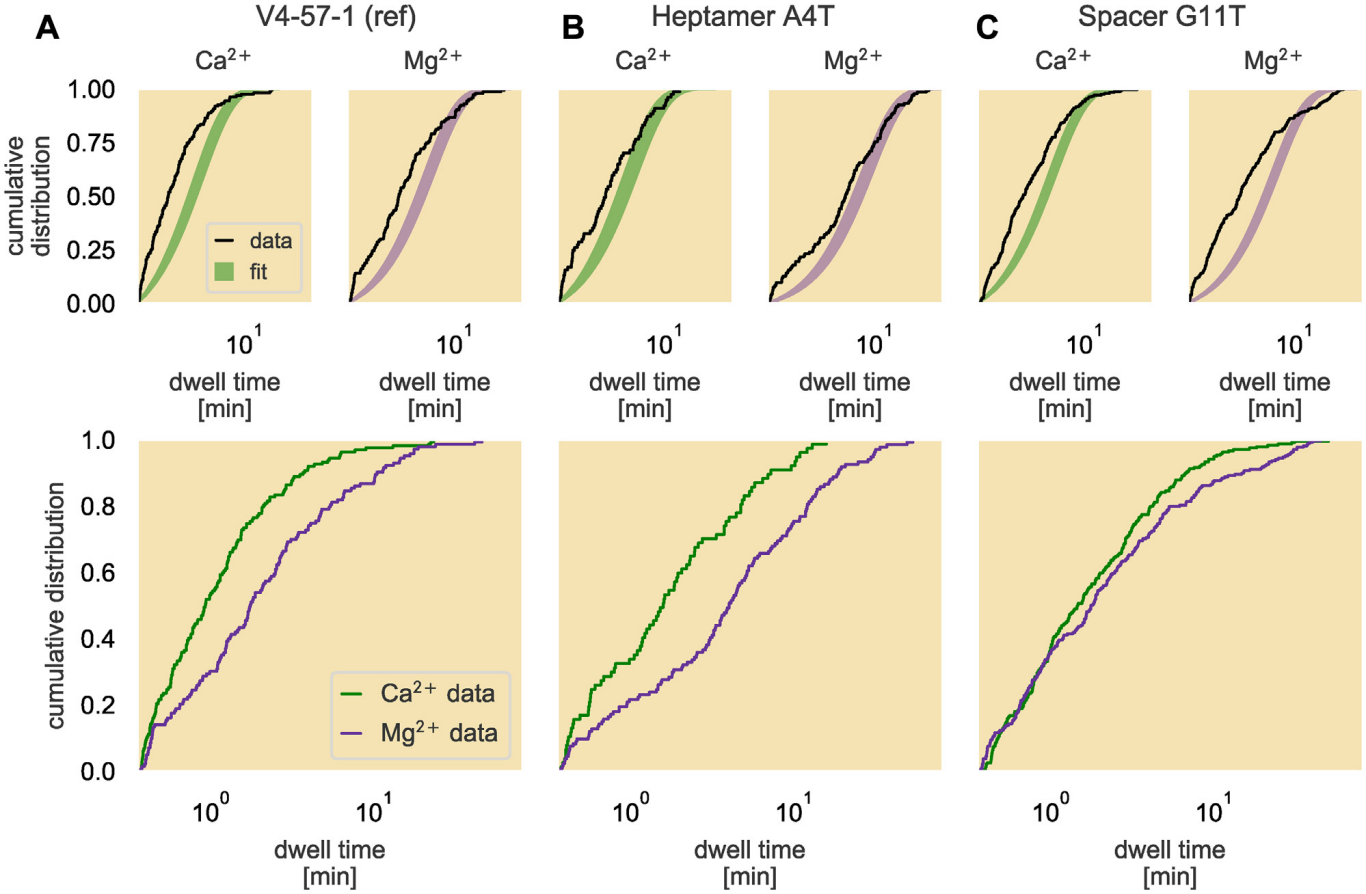
Empirical cumulative distributions of PC lifetimes with different divalent cations. The empirical cumulative dwell time distributions are plotted in black over the 95% credible region of the fit to an exponential distribution (top row) for the reference sequence (**A**), a base pair change in the heptamer region of the 12RSS (**B**) and a base pair change in the spacer region (**C**). The bottom plots show direct comparisons of the empirical cumulative dwell time distributions collected in either Ca^2+^- (green) or Mg^2+^- (purple) supplemented reaction buffer for each RSS. The dwell time axis on all plots are logarithmically scaled.

While we observe no cleavage of PCs in the Ca^2+^-based buffer, looping is as frequent, if not more frequent, than in the Mg^2+^-based buffer (see Supplementary Figure S7). However, even in the absence of nicking, the dwell times of PC events are still not in agreement with an exponential distribution (left panels of Figure 8A–C). For the dwell time distribution to defy a single-exponential form, the process of unlooping itself cannot be a Poisson process with only one kinetic rate. Extracting kinetic rates of exit from the PC state is not possible without also observing a critical, yet currently indiscernible by TPM, biochemical process between RAG and RSS. We also note that for each of the RSS variants the observed PC lifetimes are short lived compared to those in the Mg^2+^-based buffer, as can be seen in the bottom plots of Figure 8. Because Ca^2+^ does not significantly alter DNA flexibility compared to Mg^2+^ (37), our data argue that nicking itself results in a long-lasting PC. This is notable in light of recent structural evidence showing that nicking and the associated ‘flipping out’ of two bases at the RSS-gene segment junction away from their complementary bases create a fully ‘closed’ RAG–RSS binding conformation that would be predicted to improve stability of the complex (13,14). With the more stable conformation from nicking one or both RSSs, the PC state persists for longer than if RAG could not nick either RSS, which is reflected in the longer dwell time distributions when using Mg^2+^.

## DISCUSSION

Through the temporal resolution provided by TPM, we have discerned how RAG forms and cleaves the PC for a series of synthetic and endogenous RSSs. We find that the RSSs of frequently used gene segments typically do not support more efficient PC formation or cleavage than those neighboring gene segments of more modest usage. This observation is consistent with recent findings that RSS strength, as assessed by the RSS information content (RIC) algorithm (11,38–40), is only one of multiple parameters needed to be able to predict gene segment usage frequency (30,41). Furthermore, we found from analyzing single bp variations of the V4-57-1 RSS that the efficiencies of PC formation and cleavage are sensitive to single bp changes depending upon the conservation level at the respective position. We see that altering the perfectly-conserved third position of the heptamer almost completely blocked cleavage by RAG without significantly altering PC formation frequency or dwell time distribution. In contrast, certain deviations from the consensus nucleotide at the last four positions of the heptamer or in the nonamer decreased the frequency of PC formation. Finally, even though few positions of the spacer have a clear consensus nucleotide (7), formation and cleavage of the PC can still be strongly affected by a single bp change in the spacer. In fact, sequence-context effects might explain why some of these synthetic RSSs in less conserved positions of the spacer have such a strong influence on PC formation and cleavage on their own.

We asked to what extent we could account for the behavior of an endogenous RSS based on its constituent nucleotides as revealed by our synthetic RSS study. The Synthetic-Endogenous RSS Comparison interactive tool on the paper website allows one to select an endogenous RSS to reveal not only its data on PC formation, PC lifetime distributions and cleavage probability distributions, but also data for each nucleotide difference between it and the reference 12RSS through the relevant synthetic RSSs. For ease of comparing the endogenous RSS with a synthetic RSS relevant to the sequence difference, hovering the computer mouse over the nucleotide of interest in the sequence changes the color of all other relevant synthetic RSSs to gray. Although our finding that hidden molecular details in the RAG–12RSS–23RSS interaction prevent us from constructing a quantitative model that directly relates endogenous RSS behavior to the effects measured for each individual sequence deviation, these results provide several insights into the relation between RSS function and its constituent nucleotides. In particular, the data reveal a subset of RSS positions, including some in the spacer, that appear to strongly influence RAG–RSS interactions.

The synthetic RSS with the G-to-T change at spacer position 10 strongly increases the cleavage probability and also enhances PC formation (Figures 3 and 5C,D). These improvements might be due to the 5′-TG-3′ dinucleotide created by this change at spacer positions 10 and 11. Such a pyrimidine-purine (YR) pairing is inherently deformable (42) and a substantial 60° bend in the 12RSS is seen at this location in the spacer in RAG–RSS complexes (14). Hence, as noted previously (14), a YR combination at the 3′ end of the spacer in the 12RSS is favorable for DNA binding, consistent with our data. The DFL16.1-5′ RSS contains a T at spacer position 10 (Supplementary Table S1), as well as several other nucleotides in the spacer that each individually increase PC formation (see the paper website), but this RSS exhibits inefficient PC formation (Figure 6A). Because spacer position 11 is also a T in the DFL16.1-5′ RSS, the T at position 10 does not create a YR pair and instead, the last seven bp of the spacer are all pyrimidines. A spacer with such a sequence might be particularly poor at supporting the DNA distortions needed for RAG–12RSS binding. This example of the importance of sequence context in determining how a particular bp will influence RSS function supports a concept borne out of the development of the RIC algorithm (11,38,40).

The contributions that coding flanks make to RAG–RSS dynamics (13) are important considerations to quantitatively model the RAG–DNA interactions, as each endogenous RSS neighbors a different coding flank. We attributed the low cleavage probability of the V6-15 RSS to the T immediately adjacent to the RSS in the coding flank, which has been shown to be detrimental to recombination efficiency (25–27). Because the other endogenous RSSs studied are rich in C and A nucleotides in the two bp adjacent to the heptamer, we compared data for two pairs of DNA constructs that differed only in coding flank sequence. One comparison involves the substrate containing the coding flank sequence used on the V4-57-1 RSS (5′-GTCGAC) and a substrate with a C-to-A change adjacent to the heptamer (5′-GTCGAA). The other pair is the V4-55 endogenous RSS substrate and the synthetic RSS substrate containing a C-to-A alteration at spacer position 1, where, fortuitously, the RSSs are identical and the coding flanks differ by five base pairs (5′-CACCCA for V4-55 and 5′-GTCGAC for the synthetic RSS). In both cases, the looping frequencies, PC lifetime distributions, and cutting probability distributions are similar for the respective pairs, arguing that these coding flank differences contribute little to the overall RAG–RSS reaction (see Supplementary Figures S8 and S9). Hence, coding flank differences present in all of the endogenous RSS substrates analyzed here, with the exception of the V6-15 RSS, are unlikely to have a strong influence on RAG–RSS dynamics. However, a more extensive examination of coding flank, particularly for G- and T-rich sequences, in a dynamic experimental method such as TPM will help to shed light on the extent to which these RSS-adjacent sequences influence the various steps of V(D)J recombination.

The V5-43 12RSS has a low level of PC formation, likely because of its C-to-T change at nonamer position 8, while its poor cutting probability can be attributed to a collection of sequence changes that reduce cleavage probability. The low frequency of PC formation with the V9-120 and V6-15 RSSs is likely driven primarily by the A-to-T change at nonamer position 4, with additional negative contributions coming from altering the reference spacer. And the DFL16.1-3′ RSS, which supported the highest frequency of PC formation across all RSSs studied, differs from the reference RSS at the fourth and sixth positions of the spacer that each in their own synthetic RSSs strongly stimulated PC formation. These findings support the important conclusion that spacer sequence can influence RSS synapsis by RAG.

We find that the DFL16.1-5′ RSS is much less competent for PC formation and cleavage than the DFL16.1-3′ RSS. Weaker activity of the 5′ RSS compared to the 3′ RSS is consistent with the results of recombination assays performed using plasmid substrates in cells (28,29) and for chromosomal recombination when DFL16.1 was placed ≈700 bp from its *Igh* J gene segment partner, J_H_1 (24). However, when assayed in their natural location over 50 kb from the J_H_ gene segments, the two RSSs support roughly equal levels of recombination as long as they are in the same orientation relative to the J_H_ 23RSSs (24). The existing data argue that the DFL16.1-5′ RSS is intrinsically less active for recombination than the DFL16.1-3′ RSS, but this difference can be minimized over large chromosomal distances when chromatin ‘scanning’ by RAG is the dominant mechanism for bringing RSSs together to form the PC (24,43). Such scanning requires that a RAG-single RSS complex be able to either bind and then release, or else entirely skip over, proximal partner RSSs to be able to recombine with more distal RSSs. Our findings relating RSS sequence to the efficiencies of PC formation and cleavage within the PC provide a valuable resource for considering how RSS sequence might influence the scanning process.

Our study of both synthetic and endogenous RSSs explains the low usage of the V8-18 gene segment in the *Ig*κ repertoire and further highlights the strong impact that can be exerted from a single nucleotide change to an RSS. The V8-18 RSS contributes to inefficient PC formation and further interrogating each sequence mismatch between the V8-18 and reference RSSs revealed that its T-to-A alteration at heptamer position 6 is sufficient to virtually abrogate PC formation. This effect on gene segment selection may not be unique to the V8-18 gene segment: both the V1-131 and V8-26 gene segments have the same T-to-A deviation from the consensus heptamer position 6, and neither is used in recombination (5). This deviation from consensus further provides a mechanistic explanation for why the VκA2b gene segment is underutilized in the antibody repertoire of Navajos, which in turn has been proposed to account for the high susceptibility of Navajos and several genetically related Native American groups to *Haemophilus influenza* type b infection (44). The VκA2b RSS differs in sequence from the more common and efficiently recombined VκA2a RSS by a single T-to-A change at heptamer position 6 (44–46). We conclude that the inefficient recombination caused by this alteration is due to a defect in PC formation and suggest that any gene segment whose RSS contains an A at the sixth position of the heptamer will recombine poorly. Consistent with this, A is almost never observed at the sixth position of the heptamer in either the 12- or 23RSS (7).

From the length control of cytoskeletal filaments (47) to the partitioning of molecules in cellular division (48), distributions offer a mechanistic window into constraints on the class of permissible quantitative models. In the case of our efforts to calculate kinetic rates of RAG–RSS dynamics using the PC lifetime distribution, we discerned two interesting findings on the nature of the interaction. Upon first applying our fitting procedure to determine the rates of unbinding and cleavage, we learned that at least one of these two processes did not behave as a simple Poisson process. Even though the exceptionally long dwell times that contribute to the extended tail in the ECDFs may be accounted for in part by the occasional ‘dead-end’ PC where the purified RAG loses function but remains bound to the PC, the different concavities between the ECDFs and theoretical exponential distribution at the earlier lifetimes as shown in Figure 7 suggest a biochemical process involving RAG and RSS that violates a Poisson process. Thinking that our inability to detect nicking was the culprit, we examined the rate of unlooping in the absence of nicking by using Ca^2+^ instead of Mg^2+^ in our reactions. Here, our finding that the PC lifetimes were not exponential for any of the studied RSSs told us that the dwell time distribution convolves other time-sensitive processes with unlooping and cleavage. These Ca^2+^ results suggest that the PC state may have multiple conformations like the *lac* repressor (49) in that the two RAG1/2 dimers may have multiple states, or that binding to the heptamer and to the nonamer on each RSS are actually separate sequential processes. One possible source of distinct conformations is the dramatic 180° rotation of the DNA that must occur prior to nicking. Rotated and unrotated configurations of un-nicked RSSs have been identified in recent structural studies (14,15,50), but would be indistinguishable in the TPM assay. Despite these challenges to obtaining a quantitative description, our data demonstrate that nicking of an RSS is not a prerequisite for RAG to form the PC state, consistent with previous gel shift analyses performed either in Ca^2+^ or with RAG mutants lacking catalytic activity (51–54). In addition, our findings demonstrate that PCs formed in the presence of Mg^2+^, which allows for RSS nicking, are longer lived than those formed in the presence of Ca^2+^, extending previous findings made with RAG bound to single RSSs (35). While we cannot rule out the possibility that Ca^2+^ perturbs features of the PC other than the ability to undergo nicking, the inference that PCs containing nicked RSS(s) are more stable than those containing intact RSSs is consistent with the formation of a fully ‘closed’ PC conformation after nicking in which additional protein–protein and protein–DNA interactions are observed that should further stabilize the complex (13,14,50). To our knowledge, this study is the first attempt to obtain kinetic rates of unlooping from and cutting of the PC and reveals that there are still key details in the reaction whose temporal behavior have not been observed but ultimately disqualify the PC lifetime distribution from obeying a Poisson process with a single kinetic rate.

The work presented here leaves open several questions about RAG–RSS dynamics. Although our TPM assay detects PC formation and cleavage, it does not detect nicking, preventing us from determining how the RSSs studied influence the rate of nicking or when nicking occurs relative to PC formation. Even without nicking, we see that the unlooping dynamics behave differently from a simple Poisson process. This result suggests a need for an experimental method such as single-molecule FRET (55) that can detect such subtle conformational changes that occur between RAG and the RSS. Finally, we have left the 23RSS unchanged in this study, but it is possible that the trends that we see for our synthetic or endogenous 12RSSs may change with a different partner RSS and shed more light on the ‘beyond 12/23 rule’ (11,56,57). Ultimately, these finer details in the RAG–RSS interaction can provide a more complete kinetic description of the initial phases of V(D)J recombination. While we changed the 12RSS sequence in this work, the TPM assay in principle allows us to titrate other parameters, such as the distance between RSSs, or introduce more biochemical players to better contextualize our work in the bigger picture of recombination *in vivo*.

## DATA AVAILABILITY

All data and code are publicly available. Due to their large volume, raw image files can be obtained upon request. Preprocessed image data can be downloaded from CaltechDATA research data repository under the DOI:10.22002/D1.1288. Processed data files, Matlab, and Python code used in this work can be downloaded either from the paper website or on the dedicated GitHub repository (DOI:10.5281/zenodo.3463570).

## Supplementary Material

gkaa418_Supplemental_Files

## Notes

Present address: Nathan M. Belliveau, Howard Hughes Medical Institute and Department of Biology, University of Washington, Seattle, WA 98195, USA.
