# Supplementary material for: Sequence-dependent dynamics of synthetic and endogenous RSSs in V(D)J recombination
Source: Nucleic Acids Res. 2020 May 25;48(12):6726–39. doi: 10.1093/nar/gkaa418 (PMC7337519; doi:10.1093/nar/gkaa418)
Supplement: gkaa418_Supplemental_Files [file gkaa418_supplemental_files.zip › cutting_probability_model_explorer.html]

Bokeh Plot
